# Cortical Hemodynamic Responses Under Focused Ultrasound Stimulation Using Real-Time Laser Speckle Contrast Imaging

**DOI:** 10.3389/fnins.2018.00269

**Published:** 2018-04-23

**Authors:** Yi Yuan, Yanchao Zhao, Hongshuai Jia, Mengyang Liu, Shuo Hu, Yingwei Li, Xiaoli Li

**Affiliations:** ^1^Institute of Electrical Engineering, Yanshan University, Qinhuangdao, China; ^2^School of Clinical Medicine, North China University of Science and Technology, Tangshan, China; ^3^Center for Medical Physics and Biomedical Engineering, Medical University of Vienna, Vienna, Austria; ^4^Institute of Information Engineering, Yanshan University, Qinhuangdao, China; ^5^State Key Laboratory of Cognitive Neuroscience and Learning and IDG, McGovern Institute for Brain Research, Beijing Normal University, Beijing, China

**Keywords:** focused ultrasound stimulation, laser speckle contrast imaging, cerebral blood flow, rat, real-time

## Abstract

Although there is increasing use of focused ultrasound stimulation (FUS) in brain studies, the real-time changes of the cerebral blood flow (CBF) due to FUS remain unclear. In this study, we developed a novel scheme combining FUS and laser speckle contrast imaging, which can be used to measure the CBF caused by FUS in real time. The results showed that the change of CBF increased from 0 to 30 s and reached up to the maximum of 115.1 ± 6.5% at 30 s and then decreased gradually from 30 to 60 s. This study demonstrates that FUS was able to increase CBF and alter cortical hemodynamic responses, which indicates that FUS is a potential non-invasive method to study ischemic stroke rehabilitation.

## Introduction

In recent years, focused ultrasound stimulation (FUS) has been rapidly developed for neuromodulation (Naor et al., 2016). The biophysical effects of low intensity US is still unknown and several hypotheses are under investigation. Three potential mechanisms of ultrasound neuromodulation were proposed in previous studies, including soliton hypothesis (Petrov, 1975), flexoelectricity hypothesis (Heimburg and Jackson, 2005) and intramembrane cavitation hypothesis (Plaksin et al., 2014). The advantages of FUS include high stimulation depth and high spatial resolution comparing to transcranial magnetic stimulation (Bystritsky et al., 2011; Bystritsky and Korb, 2015). Previous studies have demonstrated that FUS can modulate the brain activity of animals and humans. Tufail et al. investigated the effect of transcranial ultrasound stimulation (TUS) on neuronal activity in mouse motor cortex and intact hippocampus. They found that ultrasound was able to modulate neuronal activity, evoke motor behaviors and alter synchronous oscillations (Tufail et al., 2010). Yoo et al. applied TUS to stimulate the thalamus of anesthetized rats and verified that TUS can reduce the time to emergence of voluntary movement from intraperitoneal ketamine-xylazine anesthesia (Yoo et al., 2011). Yuan et al. stimulated the rat hippocampus with low-intensity TUS and found that ultrasound can modulate power spectrum and phase-amplitude coupling of neuronal oscillations (Yuan et al., 2015, 2016), Yu et al. performed electrophysiological source imaging of TUS-induced rat brain activity and analyzed event-related potentials in time, frequency, and spatial domains, and found that neuronal activation was correlated to TUS intensity and sonication duration (Yu et al., 2016). Hakimova et al. found that TUS has the ability to inhibit acute seizure activity (Hakimova et al., 2015). Hameroff et al. used TUS to affect human mental states. They found that mood and global effect were improved 10 min and 40 min by TUS compared with placebo (Hameroff et al., 2013). Legon et al. used TUS to modulate primary somatosensory cortex on sensory-evoked brain activity and sensory discrimination abilities. The results showed that TUS significantly attenuated the amplitudes of somatosensory evoked potentials elicited by median nerve stimulation and significantly modulated the spectral content of sensory-evoked brain oscillations (Legon et al., 2014).

In this work, we expect TUS to cause a cerebral blood flow (CBF) enhancement so that it can be used for protecting the brain from ischemic stroke. Previously, Guo et al. combined pulsed transcranial ultrasound stimulation (pTUS) and laser speckle imaging to study the CBF induced by ultrasound. They found that pTUS can be used to improve the ischemic cortex after a distal middle cerebral artery occlusion (Guo et al., 2015). In another study, Dunn et al. demonstrated that the CBF can be increased during neuronal excitation of the rat somatosensory cortex (Dunn et al., 2005). Altland et al. demonstrated that low-intensity ultrasound can enhance the activity of nitric oxide synthase in vascular endothelial cells and then increase the synthesis of nitric oxide, thereby promoting the perfusion of ischemic muscle tissue (Altland et al., 2004). From these studies, we can infer that low-intensity ultrasound may protect the brain from ischemic stroke by enhancing of the blood flow in the damaged areas of the brain.

However, due to technical limitations, CBF with pTUS in the stroke group cannot be detected simultaneously and completely at the duration of stimulation because the collimator of the pTUS was perpendicularly fixed above the cortex in the stroke group (Guo et al., 2015). To solve this problem, we built a novel system to make ultrasound stimulation and laser speckle contrast imaging on a coaxial line, so it can detect hemodynamic responses due to FUS in real time. The hollow focused ultrasound transducer used in the system not only transmitted ultrasound to the brain tissue for neuromodulation but also transmitted light for laser speckle contrast imaging to detect hemodynamics simultaneously.

## Materials and methods

### Experimental setup for FUS and laser speckle contrast imaging

**Figure 1:**
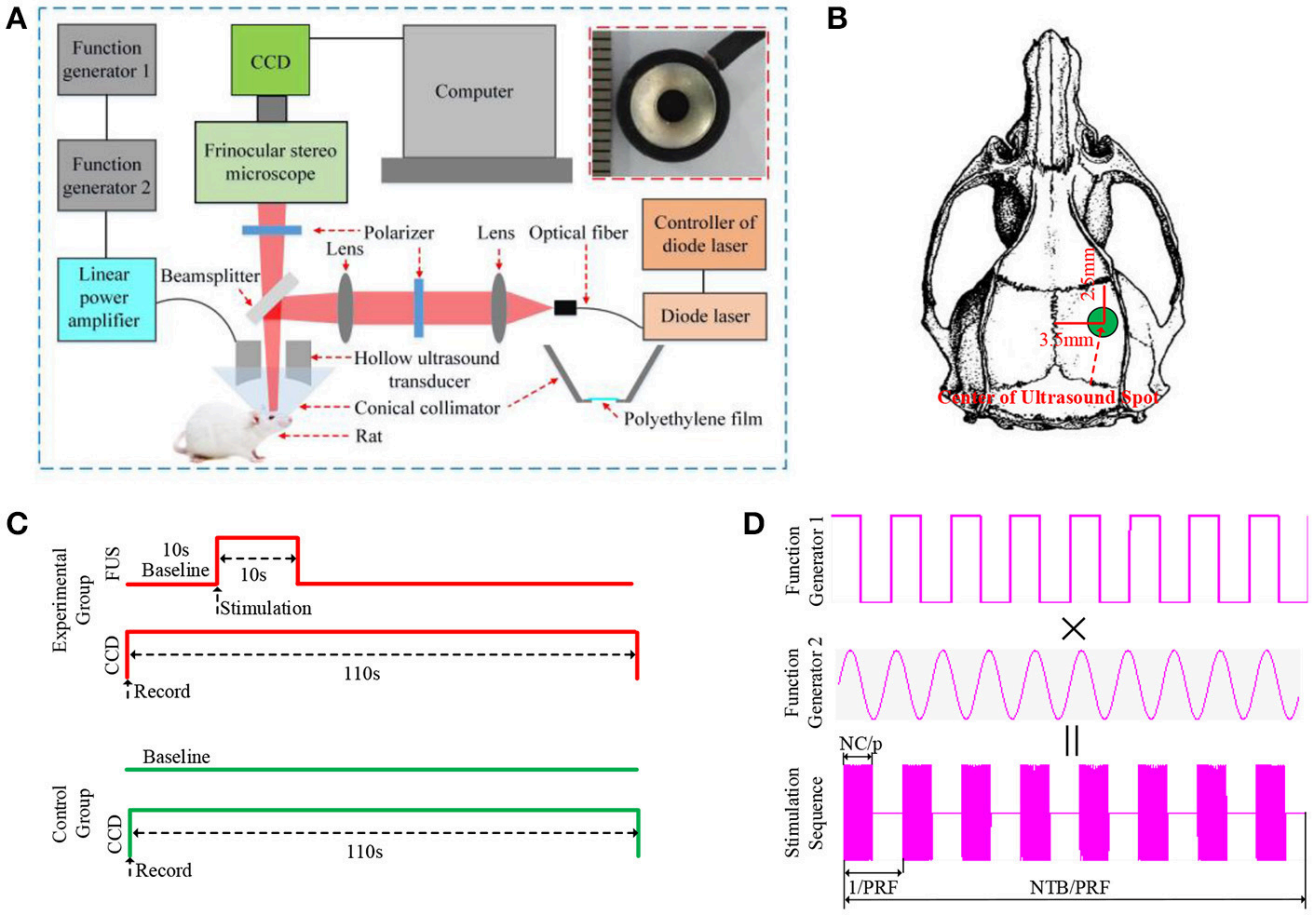
**(A)** The schematic of the experimental setup. It includes two parts: the ultrasound stimulation system and the laser speckle contrast imaging system. **(B)** the position of ultrasound stimulation, the anteroposterior (AP:3.5 mm, ML: 2.5 mm). **(C)** The sequence diagram of experiment, the recording time of CCD is 110 s (baseline 10 s, FUS 10 s, after FUS 90 s). **(D)** the sequence diagram of pulse ultrasound, PRF, pulse repetition frequency; NTB, number of tone burst; NC/p, number cycles of per pulse; PRF = 1 kHz, NTB = 300, NC/p = 1,450.

The schematic of the experimental setup is shown in Figure 1A. It includes two parts: the ultrasound stimulation system and the laser speckle contrast imaging system. In the ultrasound stimulation system, the bursts of pulsed waves were generated by two connected arbitrary function generators (AFG3022C, Tektronix, USA). The first function generator generated square waves to trigger the operation of the second function generator and was used to control the pulse repetition frequency (PRF), stimulation duration (SD), number of tone burst (NTB) and inter-stimulus interval (ISI). The second function generator was used to control the ultrasound fundamental frequency (FF), number of cycle per pulse (NC/p), and acoustic intensity (AI). The generated sinusoidal waves were then amplified by a linear power amplifier (240L, ENI Inc., USA) before being transmitted to the hollow ultrasound transducer. The customized hollow ultrasound transducer (QN8-30C/8-3, Siansonic Technology Co., Ltd., China) fixed in a fixture of the laser speckle contrast imaging system had an inner diameter, an outer diameter and a height of 3 mm, 11 mm and 20 mm, respectively. The central frequency, bandwidth and focal length of the transducer were 2.9 MHz, 60% and 9 mm, respectively. A conical cone collimator made of transparent plastic as shown in the lower right corner of Figure 1A fixed (shown in the top of Figure 1A) in the fixture and filled with water was connected to the transducer and the brain tissue. The transducer was immersed in the water-filled collimator. The water and the tissue were separated by a transparent polyethylene film. The transparent ultrasonic coupling liquid was used as a coupling layer between the polyethylene film and the tissue.

In laser speckle contrast imaging, a diode laser (MW-SGX-635; 635 nm, 20 mW; Leishi, China) beam coupled with a 600-nm diameter silica optical fiber was used as the light source. The light from the optical fiber passed through the first lens (LA1251-A, Thorlabs, U.S.A) to form an approximately parallel beam. Then it passed through the first polarizer (LPNIRE100-B, Thorlabs, U.S.A) and was focused by the second lens (LA1251-A, Thorlabs, U.S.A). The focused light was reflected by a beam splitter (BSW29R, 50:50, Thorlabs, U.S.A) and passed through the hole of the hollow focused ultrasound transducer to illuminate the brain tissue. The scattering light from the brain tissues and blood vessels passed through the hole of the transducer, the beam splitter, the second polarizer (LPNIRE100-B, Thorlabs, U.S.A) and a trinocular stereo microscope (XTL-165, Phenix, China) before being recorded by a CCD camera (CM3-U3-28S4M-CS, 12bit, Point Gray, Canada). There was an angle of 45 degrees between the beamsplitter and the light. The angle between the two polarizers was 90 degrees to eliminate the effects of the reflected light from the tissue surface on laser speckle contrast imaging. The CCD camera was mounted on the trinocular stereo microscope.

### Parameters for FUS and laser speckle contrast imaging

The sequence diagram of the pulsed ultrasound is shown in Figure 1D. The PRF and the NTB were 1 kHz and 300, respectively. The ultrasound duration and the ISI were 300 ms and 1 s, respectively. The FF and the NC/p were 2.9 MHz and 1450, respectively. We measured the ultrasound intensity by a calibrated needle-type hydrophone (HNR500, Onda, Sunnyvale, CA) at the center point of the focal spot which is located in the middle line of the hole with a distance of 9 mm to the surface of transducer element. The acoustic pressure was 0.51 MPa, which is close to the acoustic pressure of 0.53 MPa used in another study of cerebral hemodynamic change during TUS (Kim et al., 2017). We calculated the he spatial-peak and pulse-average intensity (I_sppa_) according to previous report (Kim et al., 2014) and it was ~8.93 W/cm^2^. The corresponding spatial-peak temporal-average intensity I_spta_ was ~4.47 W/cm^2^. The imaging field was tuned to 3 mm area with a resolution of 600 × 600 pixels. The laser speckle images were acquired at 21 fps (frame per second) and the exposure time was 20 ms. The sequence diagram of the experiment is shown in Figure 1C. In the experiment, the CCD camera recorded the image for 110 s, including before FUS (baseline) 10 s, FUS 10 s, after FUS 90 s. In the control group, the rats were located in the setup with the same location, but the transducer did not transmit ultrasound pulses. The CCD camera also recorded the image for 110 s.

### Animal surgery and anesthesia

A total of 30 Sprague-Dawley rats (3-month-old males, body weights ~270 g) were used in the experiment (15 rats for the experimental group, 15 rats for the control group). All procedures were carried out according to the Animal Ethics and Administrative Council of Yanshan University and Hebei Province, P.R. China. Surgical anesthesia was induced with sodium pentobarbital (3%, 5 mg/100 g, i.p.). The anesthetized rats were fixed on a stereotaxic apparatus (ST-5ND-C, Stoelting Co., U.S.A) with ear bars and a clamping device. The fur covering the rat's skull was shaved, and the skin was cleaned with a 0.9% sodium chloride physiological solution. The scalp was cut along the midline of the skull, and the subcutaneous tissue and periosteum were removed. A circular section of the skull was removed to expose the brain tissue with a radius of 3 mm. The anteroposterior (AP) and mediolateral (ML) coordinates of the center of the hole were 3.5 mm and 2.5 mm, respectively (shown in Figure 1B).

### Temporal laser speckle contrast imaging

Laser speckle contrast imaging obtained the velocity distribution information of red blood cells and thus the whole regional distribution of the velocity of fast measurements with analysis of the speckle image provided statistical comparisons (Fujii, 1994; Briers, 2001). In this study, we utilized a temporal laser speckle contrast imaging method to evaluate the changes in CBF for FUS (Li et al., 2011; Dunn, 2012; Zhang et al., 2012; Davis et al., 2016). The velocity information in the blur can be extracted and mapped with contrast using statistical arguments. In particular, laser speckle contrast (*C*_*M*_) can be defined as Li M. et al. (2009) and Li N. et al. (2009).

(1) $$\begin{array}{r} C_{M}=\frac{\langle I_{M}^{2}\rangle -{\langle I_{M}\rangle }^{2}}{{\langle I_{M}\rangle }^{2}}=\frac{\sigma_{M}^{2}}{{\langle I_{M}\rangle }^{2}} \end{array}$$

where 〈*I*_*M*_〉 and $\langle I_{M}^{2}\rangle$ are the average and the mean-square values of the time-varying speckle intensity over *M* observations. $\sigma_{M}^{2}$is the square of the standard deviation of the time-varying speckle intensity. The contrast *C*_*M*_ can be calculated over time using a time stack of images. In this case, a pixel window is moved across a time stack of *M* images to obtain the statistics leading to a temporally contrasted image.

The velocity of the scattering particles *v* and the speckle contrast *C*_*M*_ can be related through the integration time as follows (Li M. et al., 2009):

(2) $$\begin{array}{r} v=\frac{2w}{T}\frac{{\langle I_{M}\rangle }^{2}}{\langle I_{M}^{2}\rangle -{\langle I_{M}\rangle }^{2}}=\frac{2w}{T}\frac{1}{C_{M}} \end{array}$$

where *T* is the integration time and *w* is the radius of the illuminating beam.

In our trials, we obtained one contrast image from 42 original laser-speckle images. We defined the relative cerebral blood flow (rCBF) as the ratio of 1/C_*M*_ to the corresponding mean value of baseline (Li M. et al., 2009).

(3) $$\begin{array}{r} rCBF=\frac{{\left(1/C_{M}\right)}_{Sti}}{{\left(1/C_{M}\right)}_{Bas}}\times 100\% \end{array}$$

where (1/*C*_*M*_)_Sti_ is the reciprocal of laser speckle contrast with FUS, (1/*C*_*M*_)_Bas_ is the reciprocal of laser speckle contrast before the FUS.

### Calculation of the correlation coefficient

The ultrasonic wave causes the tissue to shake and induce image jitter, which in turn make the raw image shift in position. It also means that two images cannot completely coincide with TUS. A two-dimensional normalized cross-correlation algorithm was used to calculate the image correlation to determine the offset and jitter duration induced by the FUS. The correlation coefficient represents the offset of two images at the same location. The two-dimensional normalized cross-correlation algorithm is given by:

(4) $$\begin{array}{r} f\left(u,v\right)=\frac{\overset{N-1}{\underset{l=0}{\sum }}\overset{M-1}{\underset{j=0}{\sum }}S\left(u+j,v+l\right)T\left(j,l\right)}{{\left[\overset{N-1}{\underset{l=0}{\sum }}\overset{M-1}{\underset{j=0}{\sum }}S^{2}\left(u+j,v+l\right)\right]}^{\frac{1}{2}}{\left[\overset{N-1}{\underset{k=0}{\sum }}\overset{M-1}{\underset{j=0}{\sum }}T^{2}\left(j,l\right)\right]}^{\frac{1}{2}}} \end{array}$$

where *T* is the reference laser speckle image, *S* is the measured laser speckle image, and the size of image was *M*×*N*. In our study, the first image at a time of 1 s was the reference laser speckle image, the eleventh image of each second was the measured laser speckle image.

## Results

**Figure 2:**
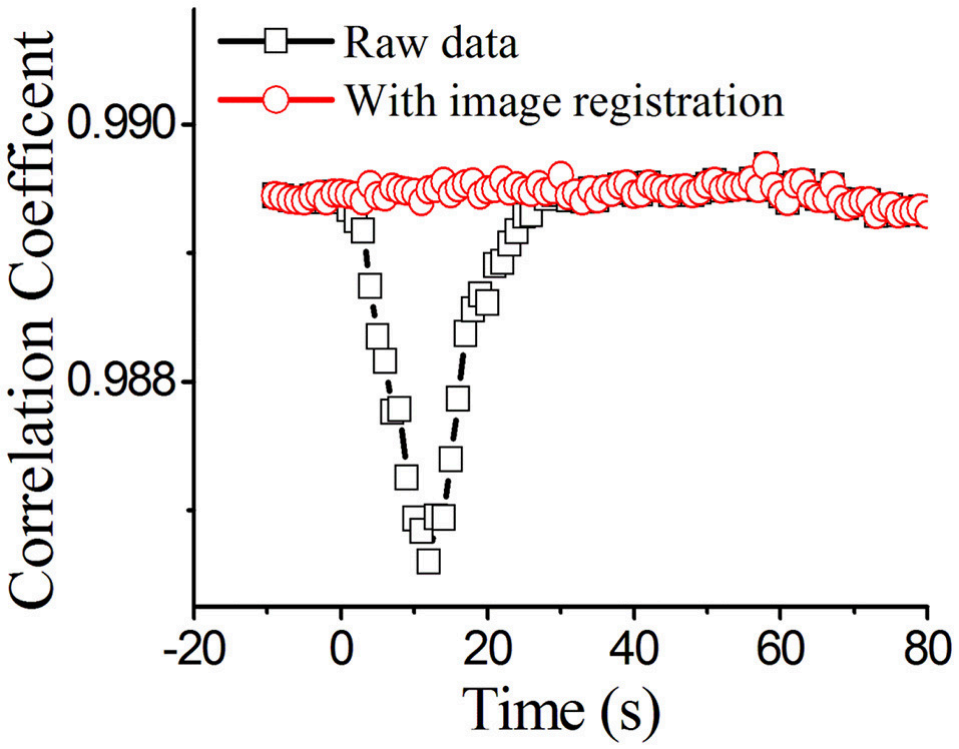
The normalized correlation coefficient. The black line is the raw data without image registration and the red line is the data with image registration.

The normalized correlation coefficient is shown in Figure 2. We found that the correlation coefficient was ~0.9895 before FUS [baseline level from −10 to 0 s. The results indicate that the brain tissue is very stable. When the brain tissue was stimulated by focused ultrasound [0–10 s], the correlation coefficient decreased. When the ultrasound was stopped, the correlation coefficient was reduced to its lowest value of 0.986. The results showed that ultrasonic vibration caused the image jitter, which changed image stability. To reduce the error caused by image jitter, image registration was done by coordinate translation according to the extracted contour of the blood vessel (Brown, 1992). We used Canny operator to detect extracted contour of the blood vessel (Canny, 1986). As shown in Figure 2, we can see that the correlation coefficient returns to the level before TUS.

**Figure 3:**
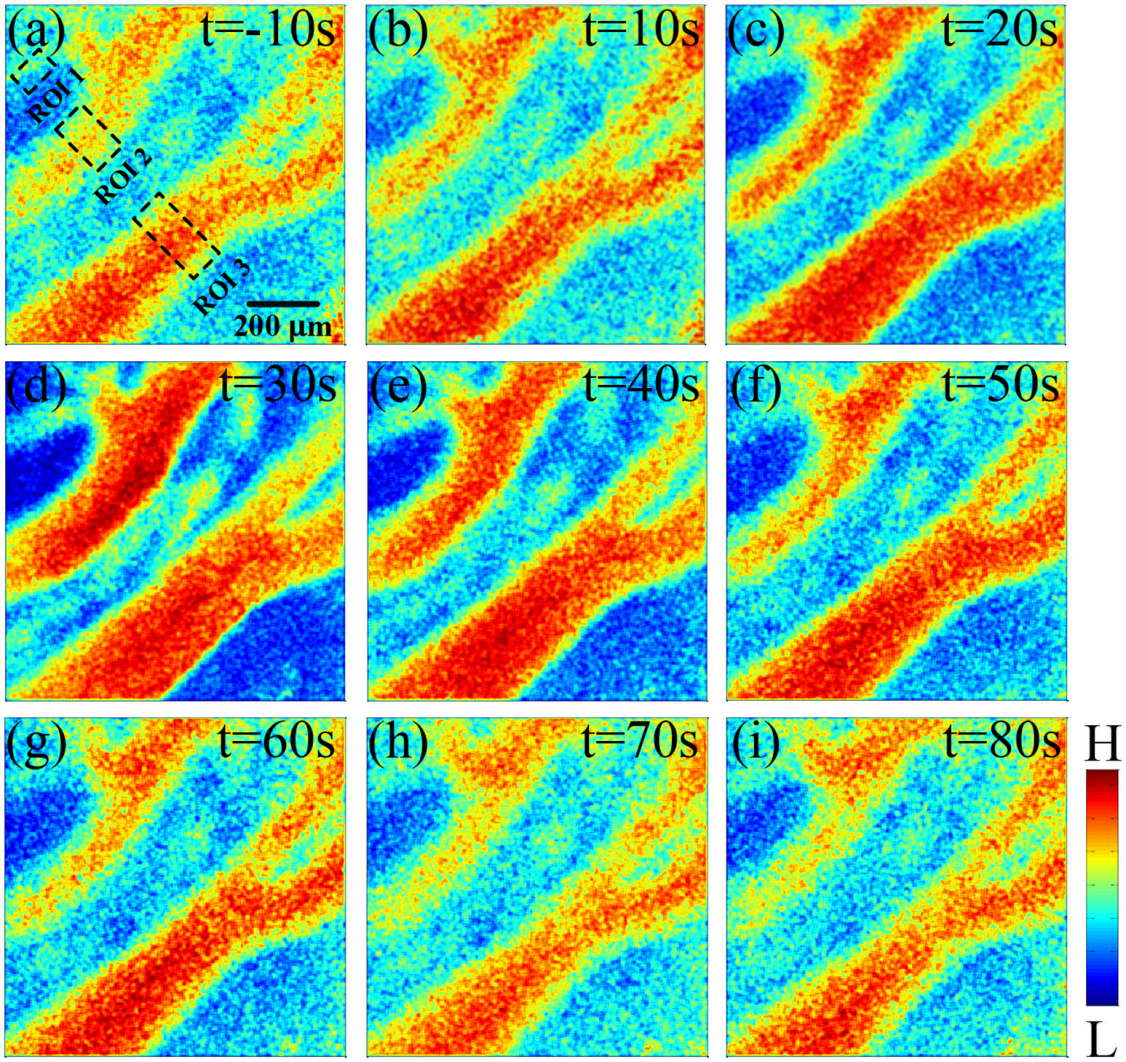
Laser speckle contrast images of stimulation group at different times. **(a)** −10 s, **(b)** 10 s, **(c)** 20 s, **(d)** 30 s, **(e)** 40 s, **(f)** 50 s, **(g)** 60 s, **(h)** 70 s, **(i)** 80 s. Compare to −10 s, the rCBF gradually increased from 10 to 30 s, and then gradually decreased from 30 to 60 s. From 60 to 80 s, the rCBF gradually recovered to a stable level and was similar to that of before stimulation.

Figure 3 shows the laser speckle contrast images of the stimulation group. In the stimulation group, compared to the results of the time of −10 s Figure 3a, the blood flow velocity significantly increased at 10 s Figure 3b. With time delay, it gradually increased from 10 to 30 s Figures 3b–d, and then gradually decreased from 30 to 60 s Figures 3d–g. From 60 to 80 s (Figures 3g–i, the blood flow velocity gradually recovered to a stable level and was similar to the state before stimulation (−10 s). These results showed that the blood flow velocity can be altered by the FUS. The change in blood flow velocity went through three stages: enhancement, weakening and recovering stabilization. The control group showed that there were no significant changes in blood flow velocity from −10 to 80 s (Figures S1a–i).

**Figure 4:**
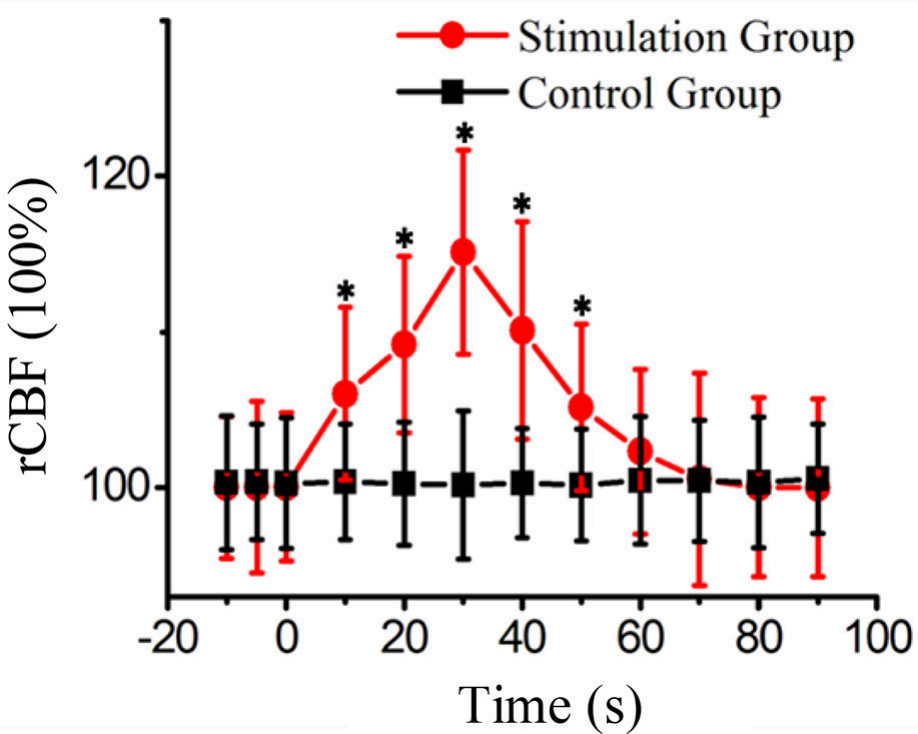
The rCBF of the stimulation group and the control group at different time points from fifteen rats. (mean±S.D. paired *t*-test with a baseline at every imaging time point, *P < 0.05).

Finally, we statistically analyzed the experimental results of 15 rats and the statistical results are shown in Figure 4. The laser speckle contrast at −10 s was set as the baseline, we calculated the rCBF at −10, −5, 0, 10, 20, 30, 40, 50, 60, 70, 80, 90 s. The corresponding values of stimulation group were 100.2 ± 4.6%, 100.3 ± 5.5%, 100.5 ± 4.8%, 106.3 ± 5.5%, 109.2 ± 5.7%, 115.1 ± 6.5%, 110.1 ± 6.9%, 105.1 ± 5.3%, 102.3 ± 5.3%. 100.5 ± 6.8%, 100.1 ± 5.7%, and 100.1 ± 5.8%, respectively (*N* = 15, mean±S.D. paired *t*-test with a baseline at every imaging time point, ^*^P < 0.05). The corresponding values of the control group were 100.3 ± 4.3%, 100.4 ± 3.7%, 100.3 ± 4.2%, 100.4 ± 3.7%, 100.3 ± 4.0%, 100.2 ± 4.8%, 100.3 ± 3.5%, 100.2 ± 3.6%, 100.5 ± 4.1%, 100.5 ± 4.0%, 100.3 ± 4.2%, and 100.6 ± 3.5%.

## Discussion

We found that the correlation coefficient between the images before FUS was ~0.9895[−10–0 s], which was not very close to 1. There were two reasons for this phenomenon. First, the random system noise caused the pixel values of each picture to be different. Second, the breathing and heartbeat of the anesthetized rats induced motion artifacts that led to different values for each pixel. In the experiment, these two conditions existed objectively and could not be avoided. To solve this problem, we maintained consistent experimental conditions, measured the blood flow of the rats and calculated the mean values of rCBF to reduce the error.

After qualitatively analyzing the laser speckle contrast images from one rat, it can be seen that FUS can enhance the CBF at the stimulated region. The blood flow reached the maximum value at time of 30 s, and then the blood flow decreased. Finally, we analyzed the rCBF for 15 rats and found that there was no obvious change of rCBF in the control group. In the stimulation group, the rCBF had no significant change at −5 and 0 s. The rCBF increased from 0 to 30 s and reached the maximum at 30 s and then decreased gradually until 60 s closing in to the baseline value. Under the current ultrasound parameters, the rCBF of 15 rats was 115.1 ± 6.5%. If the ultrasound parameters were changed, the value of rCBF may be altered. Therefore, we will detect the CBF with FUS under different ultrasound parameters in the future.

In our study, we combined FUS and laser speckle imaging to investigate the CBF induced by FUS. LSCI technology can obtain regional blood-flow distributions without scanning (Li M. et al., 2009). LSCI also offers many advantages over other traditional methods including laser Doppler flowmetry or function magnetic response imaging, such as high spatial and temporal resolutions, imaging without contrast agents and real-time imaging. LSCI has seen wide use in nerve blood-flow imaging and is especially suitable to the study of neural activity and hemodynamics.

**Figure 5:**
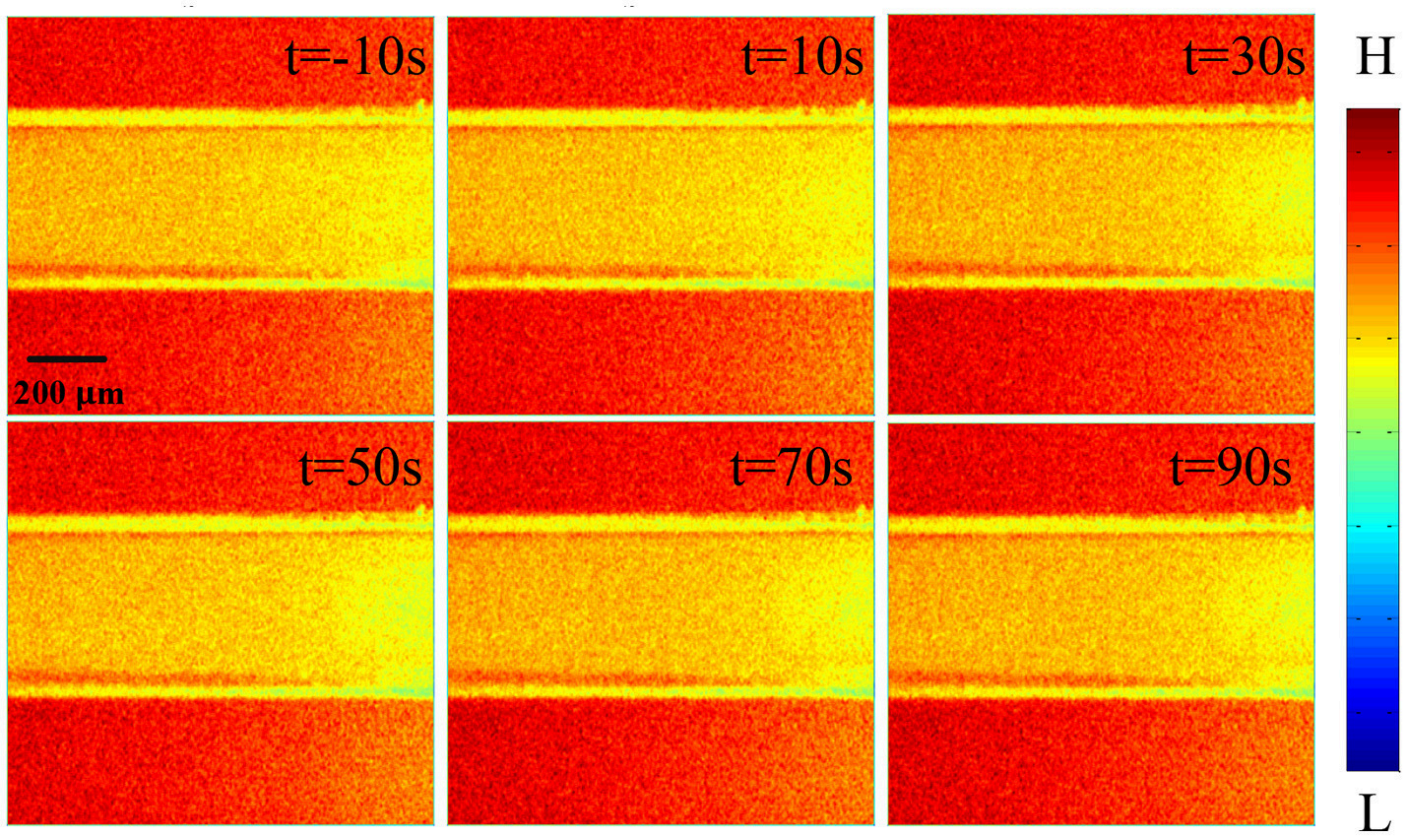
Laser speckle contrast image of isolated blood with FUS. The relative flow of isolated blood has no change with TUS. The ultrasound parameters: FF = 2.9 MHz, PRF = 1 kHz, NTB = 300, NC/p = 1450, ultrasound pressure = 0.51 MPa.

As we know, when the ultrasound transmits to brain, the tissue and blood vessel will receive the acoustic radiation force. To demonstrate the effect of acoustic radiation force on blood vessel mechanical displacement, we measured and compared the positions of the centerlines of blood vessels in all results (*N* = 15), finding that the positions of blood vessel did not change. When ultrasound and tissue (including tissue and blood) interact, there is also ultrasound scattering in tissue. In order to prove whether the ultrasound waves move red blood cells and the ultrasound scattering cause decorrelation of speckles. We collected blood from rats and dissolved them with heparin sodium. The blood was pushed into a glass capillary tube with an inner diameter of 0.4 mm by a peristaltic pump (HL-2B, Shanghai Chitang Electronic Co. LTD, China). The velocity of the blood is 1 mm/s. The wall thickness of the glass capillary was 50 μm. The glass made by quartz has an acoustic impedance of 1.81 MRayl. The blood was irradiated by ultrasound and the raw laser speckle images were recorded. The experiment process was the same as that used in the animal experiments. The FF, PRF, NTB, NC/p and ultrasound pressure are 2.9 MHz, 1 kHz, 300, 1,450, and 0.51 MPa. As showed in Figure 5. we find that there is no change of speckle image and the flow of isolated blood has no change with ultrasound stimulation. We demonstrate that the ultrasound scattering does not affect the laser speckle imaging and can see no effect of ultrasound wave induced red blood cell movement. In the experiment, we expect sufficient transmission of the ultrasound wave through the glass capillary wall, so that ultrasound can sufficiently interact with the red blood cells *in vitro*. As we know, the frequency dependent absorption for ultrasound waves determines the penetration depth of ultrasound in medium. The higher the frequency, the lower the ultrasound penetration depth in the same medium. At the same time, we also know that the thickness of the glass capillary wall affects the attenuation of ultrasound waves. The attenuation increases with increasing thickness of the glass capillary wall. Therefore, we can increase the penetration depth by reducing the frequency of ultrasound or the thickness of the glass wall.

We think there may be two reasons for the increase of CBF induced by FUS. Firstly, the ultrasound stimulation induces neuron activity that in turn affects the CBF. This effect can be indicated by Dunn et al.'s work, in which they demonstrated that the CBF can be increased during neuronal excitation of the rat somatosensory cortex (Dunn et al., 2005). Secondly, FUS can enhance the activity of nitric oxide synthase in vascular endothelial cells and increase the synthesis of nitric oxide. The nitric oxide synthase can cause vasodilatation and the synthesis of nitric oxide can accelerate blood infusion (Altland et al., 2004). To demonstrate that FUS can cause vasodilation, we used the method introduced by Aylward et al. (1996) and Li N. et al. (2009) to measure the diameter of blood vessels at measurement location with and without FUS in 15 rats. The segmentation method based on the ridge tracking approach was used to segment a small length of the vessel (Aylward et al., 1996). The mean value of the detected diameters at each pixel on the vessel-centerline was then taken (Li N. et al., 2009). Since the initial values of the diameters of the blood vessels are different, we chose the relative change (diameter at different time points divided by baseline *t* = −10 s) as the evaluating indicator. The corresponding values of relative changes of diameter at 0, 10, 20, 30, 40, 50, and 60 s were 100.1 ± 0.6%, 103.3 ± 1.2%, 106.5 ± 1.3%^*^, 109.3 ± 1.2%^*^, 106.2 ± 1.1%^*^, 104.1 ± 0.8%^*^, and 101.1 ± 0.7%, respectively (*N* = 15, mean±S.D. paired *t*-test, ^*^P < 0.05). We can see that the diameter significantly increased at the time of *t* = 30 s, and then gradually decreased from 30 to 60 s. The results demonstrate that TUS not only increases CBF but also causes vasodilatation.

In our experiment, the FF of ultrasound is 2.9 MHz. The PRF, NTB, NC/p, and SD were 1 kHz, 300, 1,450 and 10 s, respectively. The I_sppa_ and I_spta_ were ~8.93 W/cm^2^ and ~4.47 W/cm^2^. In Guo et al.'s paper, the FF of ultrasound is 0.5 MHz. The PRF, NTB, NC/p and SD were 1.5 kHz, 600–1,000, 200, and 0.4–0.67 s, respectively. I_sppa_ and I_spta_ were ~2.15 W/cm^2^ and ~43–72 mW/cm^2^. The time point corresponding to peak blood flow value is 30 s in our study. However, it is at 5 s in Guo et al.'s paper. We think the reason of the difference is that we used different ultrasound parameters.

The attenuation of ultrasound intensity in the tissue follows the formula A = A_0_exp (-α_0_*f*^*n*^*l*), where *l* is the acoustic pathlength in attenuating medium; A_0_ is the amplitude at *l* = 0; n is power of frequency dependence of α; α_0_ is a constant; f is the ultrasound frequency. As seen from this equation, the attenuation of ultrasound intensity increases with increasing ultrasound frequency. When the initial ultrasound intensity is constant, the radiation force acting on the tissue and blood vessels decrease as ultrasound frequency increases. Hameroff et al. used ultrasound with a frequency of 8 MHz to stimulate frontal-temporal cortex of human (Hameroff et al., 2013). They found that transcranial ultrasound can affect mental states of human. In the Monti et al. study, they used ultrasound with a frequency of 0.65 MHz to stimulate human thalamus for protecting disorders of consciousness after severe brain injury (Monti et al., 2016). Therefore, we think that the ultrasound frequency of 2.9 MHz in our experiment can be used for stimulating human cortex. But we are not sure it can reach the thalamus, because 2.9 MHz is much higher than 0.65 MHz. In our experiment, we used ultrasound with a frequency of 2.9 MHz for basic animal research. For human experiment, we believe that a lower frequency (less than 1 MHz) would be preferred.

It is very important to estimate the thermal effects of FUS on brain tissue. The potential temperature increase due to ultrasound parameters can be estimated by the equation (Collins et al., 2004; Lee et al., 2015) Δ*T* = 2α*It*/ρ*C*_*p*_with an absorption coefficient α = 0.0175 cm^−1^, an ultrasound intensity *I* = 8.93 W/cm^2^, a FUS duration *t* = 0.3 s, a brain tissue density ρ = 1.007^*^10^3^ kg/m^3^, and a specific brain tissue heat *C*_*p*_ = 1.007^*^10^3^J^*^kg^−1°^C^−1^. Therefore, the maximum temperature enhancement induced by FUS would be 0.047°C, which is far from the temperature threshold that can induce tangible thermal bioeffects.

In summary, FUS can enhance CBF and alter cortical hemodynamic responses.

## Author contributions

YY, YZ, and XL: designed and coordinated the study. YY, YZ, HJ, SH, YL, and XL: carried out experiment and data process, and drafted the manuscript. All authors gave final approval for publication.

### Conflict of interest statement

The authors declare that the research was conducted in the absence of any commercial or financial relationships that could be construed as a potential conflict of interest.
