# Supplementary material for: Cortical Hemodynamic Responses Under Focused Ultrasound Stimulation Using Real-Time Laser Speckle Contrast Imaging
Source: Front Neurosci. 2018 Apr 23;12:269. doi: 10.3389/fnins.2018.00269 (PMC5925215; doi:10.3389/fnins.2018.00269)
Supplement: Supplementary file 1 [file Image1.PDF]

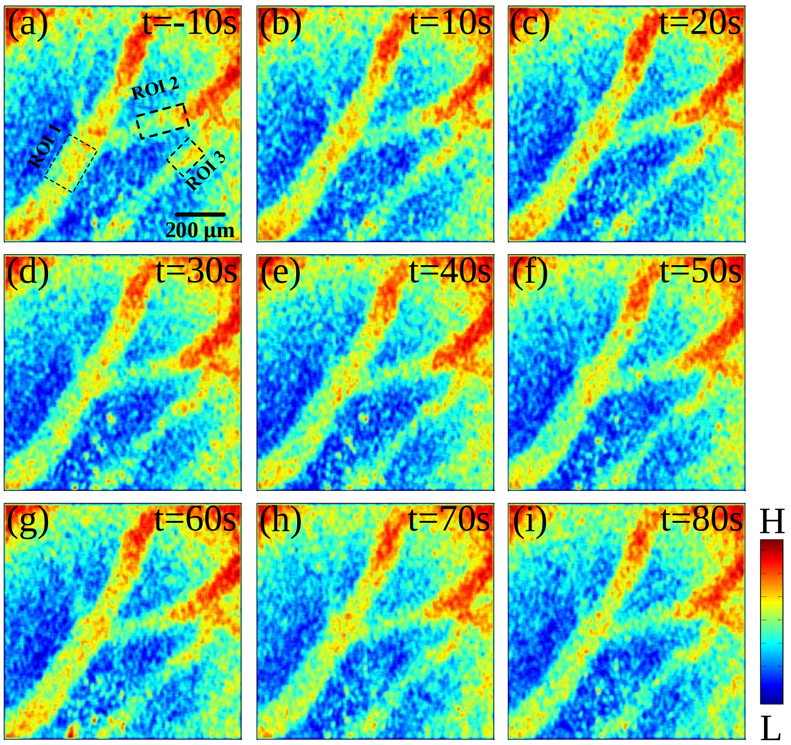

Figure. S1. Laser speckle contrast images of control group at different times. (a) -10 s, (b) 10 s, (c) 20 s, (d) 30 s, (e) 40 s, (f) 50 s, (g) 60 s, (h) 70 s, (i) 80 s.
